# Pre-clinical assay of the tissue integration and mechanical adhesion of several types of cyanoacrylate adhesives in the fixation of lightweight polypropylene meshes for abdominal hernia repair

**DOI:** 10.1371/journal.pone.0206515

**Published:** 2018-11-02

**Authors:** Gemma Pascual, Claudia Mesa-Ciller, Marta Rodríguez, Bárbara Pérez-Köhler, Verónica Gómez-Gil, Mar Fernández-Gutiérrez, Julio San Román, Juan M. Bellón

**Affiliations:** 1 Department of Medicine and Medical Specialties, Faculty of Medicine and Health Sciences, University of Alcalá, Madrid, Spain; 2 Networking Biomedical Research Center on Bioengineering, Biomaterials and Nanomedicine (CIBER-BBN), Ramón y Cajal Health Research Institute (IRYCIS), Madrid, Spain; 3 Department of Surgery, Medical and Social Sciences, Faculty of Medicine and Health Sciences, University of Alcalá, Madrid, Spain; 4 Polymer Biomaterials Group, Polymer Science and Technology Institute-Consejo Superior de Investigaciones Científicas (ICTP-CSIC), Networking Biomedical Research Center on Bioengineering, Biomaterials and Nanomedicine (CIBER-BBN), Madrid, Spain; University of Zaragoza, SPAIN

## Abstract

**Introduction:**

Lightweight (LW) polypropylene (PP) meshes better adapt to host tissue, causing less fibrosis and inflammatory responses than high-density meshes. Mesh fixation using tissue adhesives (TA) that replace conventional sutures may improve the process of hernia repair and tissue trauma. This preclinical study compares the behavior of different cyanoacrylate-based adhesives in the fixation of LW-PP meshes for hernia repair.

**Methods:**

Partial abdominal wall defects were repaired using LW-PP Optilene meshes in New Zealand rabbits. The following groups were established according to the mesh fixation method: Suture (control), Glubran 2 (n-butyl), Ifabond (n-hexyl), SafetySeal (n-butyl) and Evobond (n-octyl). At 14, 90 and 180 days after surgery, the recovered implants were examined to assess the host tissue integration, the macrophage response and the biomechanical strength.

**Results:**

All the groups showed optimal host tissue incorporation regardless of the fixation procedure. Significantly increased levels of collagen 1 and collagen 3 gene expression (p<0.001) were observed at 14 days compared to the medium- and long-term durations, where the Suture and Glubran groups showed the highest expression of collagen 1. All the adhesives increased the macrophage reaction (p<0.001) compared to sutures at all implant times. Maximal macrophage response was observed in the short-term Glubran group (p<0.01) compared to the rest of the groups. Although SafetySeal and Evobond did not reach the biomechanical resistance of sutures at 14 days, all the adhesives did reach this level in the medium- to long-term periods, providing significantly higher resistance (p<0.05).

**Conclusions:**

All the cyanoacrylates, despite inducing a significantly increased macrophage response versus sutures, showed optimal host tissue integration and long-term mechanical behavior; thus, they might be good choices for LW-PP mesh hernia repairs.

## Introduction

Tissue adhesives (TAs) have seen multiple applications in the field of surgery [1]. One of the fields in which their use has progressed the most in recent years is abdominal wall surgery, especially in relation to the surgical treatment of hernia processes [2]. In fact, one of the purposes of TAs in hernia surgery is to fix the prosthetic materials to the tissues, replacing sutures in this function [3–5]

The easy application of TAs, along with the fast application and the lesser tissue trauma that they cause, give them advantages over sutures. Sutures have been associated with the appearance of clinical symptoms of pain both in the postoperative period and in the long term, in patients undergoing inguinal hernia [6,7]. The phenomenon of nerve entrapment is among the possible causes of postoperative pain [8].

The tissue adhesives that are most widely used specifically in hernia repair have been fibrins of biological origin [9–12] and, on a smaller scale, cyanoacrylates of synthetic origin [13–15]. There is still a certain distrust of cyanoacrylates, mainly due to properties such as viscosity, polymerization time and biodegradation. All these properties can now be modified, leading to excellent results with these TAs. Toxicity from cyanoacrylates is practically non-existent, especially in those whose chemical structures are long chains, and these are authorized for use in clinical practice [16].

Regarding prosthetic materials intended for hernia repair, polypropylene in the form of a mesh is still the most used material. Polypropylene is an inert material with good tissue integration, which gives it excellent mechanical resistance properties. Depending on its structure and specifically on its porosity, there are two types of polypropylene mesh: high-density (heavyweight (HW)), with small pores and a density greater than 80 g/m2, and low-density (lightweight (LW)), with large pores and a density less than 50 g/m2 [17]. Both types of mesh are used regularly in the clinic, despite having very different mechanical characteristics [18]. The low-density or LW meshes have greater elasticity and seem to better adapt to the physiology of the abdominal wall and to the recipient tissue since they are fully accommodated to the traction of the external oblique muscles. Among other advantages offered by this type of prosthesis are the elasticity and greater distensibility of the prosthesis, which allow for non-restrictive mobility of the abdominal wall, along with optimal tolerance in the organism, minimizing the inflammatory reaction due to the rapid integration and the wide pores, which prevent the formation of seromas and hematomas. In addition, in their integrative process, these meshes cause less fibrosis and less stiffening of the tissue [19, 20]. Early tissue collagenization has also been demonstrated [21].

In previous works, we tested the biological and mechanical effectiveness of high-density meshes fixed with cyanoacrylates, obtaining mechanical fixation results similar to those when fixing with sutures [22]. In the present experimental study, taking into account the advantages offered by the low-density prostheses and to solve the additional disadvantage of their difficult fixation with sutures to the recipient tissue due to its substantial porosity, which can lead to disinsertions of the material, we designed an assay using these meshes, fixing them with both conventional commercial and experimental cyanoacrylates. As controls, the meshes were fixed with stitches. In this way, we could compare the results in terms of integration and mechanical strength.

## Materials and methods

### Experimental animals

The study protocol adhered to the ARRIVE (Animal Research: Reporting of *In Vivo* Experiments) guidelines for the publication of animal studies [23].

For the study, ninety New Zealand White rabbits, weighing between 3000 g and 3500 g, were used. The maintenance and handling of the animals throughout the study was made in accordance with the Guide for the Care and Use of Laboratory Animals of the National and European Institutes of Health (Spanish Law 6/2013, Spanish Royal Decree 53/2013, European Directive 2010/63/UE and European Convention of the Council of Europe ETS123). All the procedures done in the study were performed at the Animal Research Centre of the Universidad de Alcalá (Madrid, Spain), which is registered with the Directorate General for Agriculture of the Ministry of Economy and Technology Innovation of the Community of Madrid (ES280050001165), indicating that all facilities legally covered the needs and requirements of the research. The study protocol was approved by the Committee on the Ethics of Animal Experiments of the University of Alcalá.

### Materials used in the study

Optilene Mesh Elastic (B/Braun, Germany) was the prosthetic material used for the repair of the partial defect created in the abdominal wall. This material is a non-absorbable, lightweight (48 g/m^2^) and large-pore (3.6 x 2.8 mm) polypropylene mesh. This material has a high multidirectional elasticity so that it adapts better than the high-density meshes to the movements that occur in the abdominal wall.

The prosthesis fixation devices in this study were 2 commercial cyanoacrylate tissue adhesives with different lateral chain lengths—Glubran 2 (n-butyl-cyanoacrylate; GEM S.R.L., Italy), Ifabond (n-hexyl-cyanoacrylate; IFA Medical, France)—and 2 experimental cyanoacrylate tissue adhesives that have never been used in mesh abdominal wall repair—SafetySeal (n-butyl-cyanoacrylate; Noricum, Spain), EVOBOND 060 (n-octyl-cyanoacrylate; Tong Shen Enterprise, Taiwan). Finally, a control group was fixed with a polypropylene 4/0 monofilament and non-absorbable suture (Surgipro II; Medtronic, USA).

### Surgical technique

To minimize pre- and postoperative pain, all the animals received an analgesic dose of buprenorphine (0.05 mg / kg) (Buprecare; Divasa Farmavic, Spain), administered in their drinking water one hour before the surgical procedure as a preemptive analgesia to guarantee its effect during the procedure and at the beginning of the postoperative recovery. Intramuscular anesthesia was administered by a mixture of ketamine (70 mg/kg) (Ketolar; Parke-Davis, Spain), diazepam (1.5 mg/kg) (Valium; Roche, Spain) and chlorpromazine (1.5 mg/kg) (Largactil; Rhone-Poulenc, Spain).

**Fig 1:**
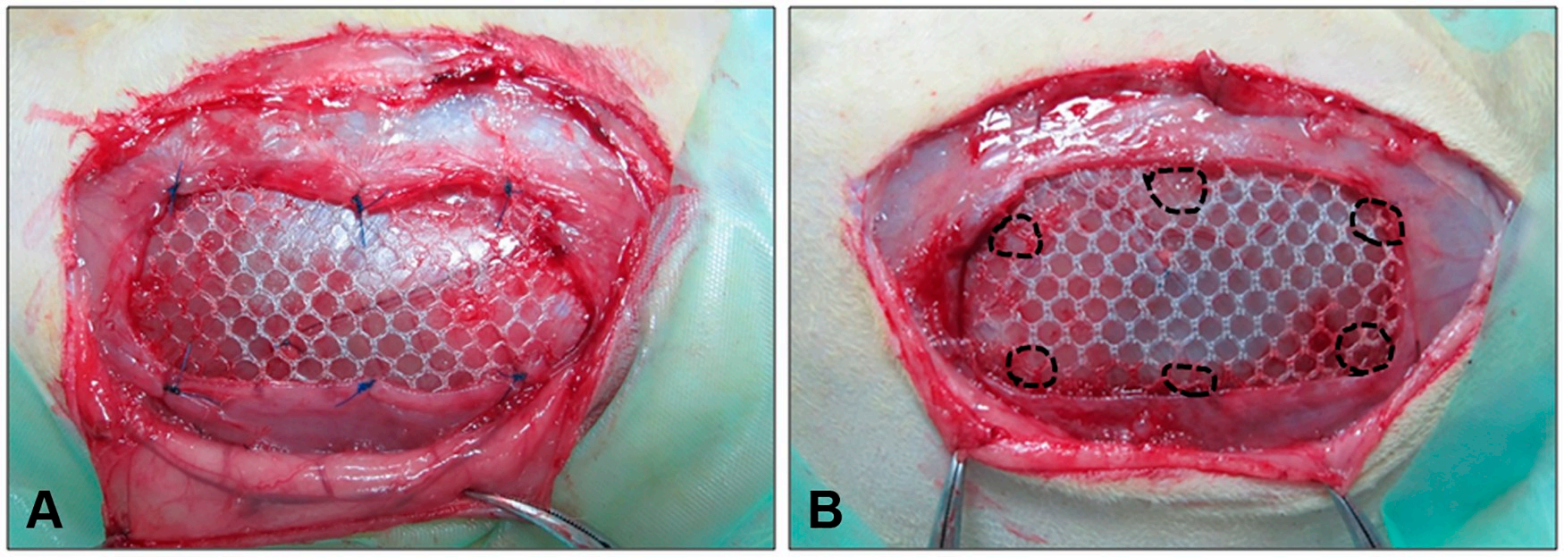
Surgical fixation of the mesh. Macroscopic images of the Optilene Mesh Elastic polypropylene mesh fixed to the edges of the defect. The fixation was made with 6 polypropylene suture points (A: control group) or with 6 drops of the specified cyanoacrylate tissue adhesive (B: experimental group) on the day of the repair surgery.

A partial defect with a size of 5 x 3 cm was created in the ventral abdominal wall, in one of the lateral sides of the linea alba, comprising the anatomical planes of the internal and external oblique muscles, respecting the transverse muscle, fascia and parietal peritoneum. Afterwards, each of the defects was repaired by placing the polypropylene lightweight Optilene Mesh Elastic and fixing it to the edges of the defect with 6 polypropylene suture points (control group) or 6 drops of the specified cyanoacrylate tissue adhesive (experimental groups) (Fig 1). Stitches or tissue adhesive drops were placed at the four corners of the implanted mesh and at the midpoints of the longer sides of the meshes. The skin was closed with a non-absorbable polypropylene 3/0 suture. Administration of 0.05 mg/kg buprenorphine in the drinking water was continued during the 3 following days to minimize pain.

At the end of each study time (14, 90 or 180 days after implantation), the animals were anesthetized and then euthanized given a lethal dose of 20% sodium pentobarbital (Dolethal; Ventoquinol S.A., France). After that, animals were monitored for the following signs: cessation of respiration and heartbeat, and pupillary dilation and unresponsiveness to light, to confirm death. Afterwards, samples of the implant with surrounding tissue were collected. Each collected sample was divided into 3 pieces, 1.5 cm wide. The 2 end strips were used for the biomechanical study, while the central strip was destined for morphological studies, immunohistochemistry and qRT-PCR.

### Experimental design

Four different study groups and the control group (n = 6 each) were established according to the fixation method:

- Group I (n = 6): Glubran 2;
- Group II (n = 6): SafetySeal;
- Group III (n = 6): Ifabond;
- Group IV (n = 6): Evobond.
- Control Group (n = 6): monofilament polypropylene suture

Each group was further subdivided by sacrifice time: 14, 90 and 180 days postimplantation.

### Morphological analysis

The samples destined for light microscopy analysis were embedded in paraffin after fixation in F-13 solution, and 5-μm-thick sections were obtained. Afterwards, the sections were deparaffinized in xylol and graded alcohols and were rehydrated and stained with hematoxylin and eosin, Masson’s trichrome (Goldner-Gabe) and sirius red. These samples were observed using a light microscope (Carl Zeiss, Germany). Sirius red staining was also observed under polarized light, which allowed observation of collagen levels and different collagen fibers orientations. Type I collagen, appears as a reddish-orange stain, while type III collagen takes on a yellowish-green shade when observed under the polarized light microscope.

### Macrophage response analysis

To assess the macrophage response to the implant and to the different fixation methods, an immunohistochemical technique was performed in paraffin-embedded sections, using a monoclonal antibody to rabbit macrophages RAM-11 (DAKO M-633, USA) in the alkaline phosphatase-labeled avidin-biotin method. The nuclei were counterstained with acid hematoxylin. For detection and quantification of the macrophage response, at least 20 microscopic fields (x200) of each sample were counted.

### Real-time reverse transcription-polymerase chain reaction (qRT-PCR)

The samples were collected and stored at -80 °C until RNA isolation. The RNA was extracted using the guanidine-phenol-chloroform isothiocyanate method with TRIzol (Invitrogen, USA). After breaking and homogenizing every tissue section, they were centrifuged, and RNA was recovered from the aqueous phase, precipitated with isopropanol and centrifuged several times with 70% alcohol to clean the RNA pellet. RNA concentrations were measured using a NanoDrop ND-100 spectrophotometer (Thermo Fisher Scientific Inc., USA) by measuring absorbances at 260 nm. RNA quality was verified with 260/280 nm and 260/230 nm ratios.

Reverse transcription (RT) was done with oligo dT primers (Amersham, USA) and the M-MLV reverse transcriptase enzyme (Invitrogen, USA) to synthesize complementary DNA (cDNA) from 50 ng/μl of total RNA. To verify the absence of genomic DNA, a negative RT without M-MLV enzyme was run.

cDNAs were diluted 1:20, and 5 μl of this dilution with 10 μl of iQ SYBR Green Supermix (Bio-Rad Laboratories, USA) and 1 μl (6 μM) of each primer (forward and reverse) were used for the qPCR in a final volume of 20 μl. This qPCR was performed in a StepOnePlus Real-Time PCR System (Applied Biosystems, USA) using the following primers: glyceraldehyde-3-phosphate dehydrogenase (GAPDH) (forward 5′-TCA CCA TCT TCC AGG AGC GA-3′ and reverse 5′-CAC AAT GCC GAA GTG GTC GT-3′), collagen 1A2 (col 1) (forward 5′-ATG GTG GCA CCC AGT TTG AA-3′ and reverse 5′-AGG TGA TGT TCT GAG AGG CG-3′) and collagen 3A1 (col 3) (forward 5′-TGC TAA GGG TGA AGT TGG AC-3′ and reverse 5′-CCG CCA GGA CTA CCA TTG TT-3′). The results of gene expression of each studied gene were normalized against GAPDH.

### Biomechanical study

The biomechanical study samples were collected in minimal essential medium (MEM) immediately after sacrifice to preserve them correctly until the tensiometric tests were performed. The uniaxial tensile strength of each sample was measured on 1.5 x 7 cm strips comprising the mesh fixed with suture or adhesive and host tissue. The tensile strength of the anchoring areas where the prosthesis was fixed to the tissue was evaluated. All the measurements were made using an INSTRON 3340 tensiometer equipped with pneumatic grips (Instron Corp., USA). The mean of the maximum strength values for the different groups were estimated in newtons (N) and the load-stretch curves (N/mm) for each sample in the different groups and study times were provided as supporting information.

### Statistical analysis

Statistical comparisons were performed using the GraphPad Prism 5 computer package for Windows (GraphPad Software Inc., USA). The Mann-Whitney *U* test was used to compare groups and time points. All the data are reported as the means ± standard error of the mean (SEM). Significance was set at *p*≤0.05.

## Results

### Macroscopic analysis

At the end of each study period, some complications were found, excluding the group where meshes were fixed with Glubran 2 cyanoacrylate, which showed no complications at any of the study times. Fourteen days after implantation, seroma and encapsulation were the most frequent complications. The group that showed the most complications in the short term was Evobond, in which 2 animals showed encapsulation, 1 infection, 1 suffered encapsulationa and seroma and the last one seroma and unintegrated mesh. Seroma was also observed in 1 of the 6 animals in the suture group and the SafetySeal group. Two animals in the SafetySeal group and 1 in the Ifabond group also showed encapsulation at this study period. Granuloma formation was seen in 1 animal in the Ifabond group (Table 1). At 90 days, two cases of infection were observed in the control and Ifabond groups, with no other complications. These complications, that are also indeed present and relatively frequent in the early postoperative period and usually resolve over time without intervention, during the process of tissue repair, were resolved until the long term where no macroscopic complications were found 180 days after surgery in any of the animals.

**Table 1:** Postoperative macroscopic complications.

Macroscopic complications	Suture			Glubran			Safetyseal			Ifabond			Evobond		
	14d	90d	180d	14d	90d	180d	14d	90d	180d	14d	90d	180d	14d	90d	180d
**Seroma**	1	0	0	0	0	0	1	0	0	0	0	0	2	0	0
**Infection**	0	1	0	0	0	0	0	0	0	0	1	0	1	0	0
**Encapsulation**	0	0	0	0	0	0	2	0	0	1	0	0	3	0	0
**Granuloma**	0	0	0	0	0	0	0	0	0	1	0	0	0	0	0
**No integration**	0	0	0	0	0	0	0	0	0	0	0	0	1	0	0

The table shows the postoperative macroscopic complications in the different groups after polypropylene lightweight mesh implantation with the different fixation method. Each box shows the number of affected animals at the different study times (14, 90 and 180 days) out of the 6 animals included in each study group. Note that in the group Evobond 14 days one animal suffered encapsulation with seroma at the same time and another one seroma and unintegrated mesh.

**Fig 2:**
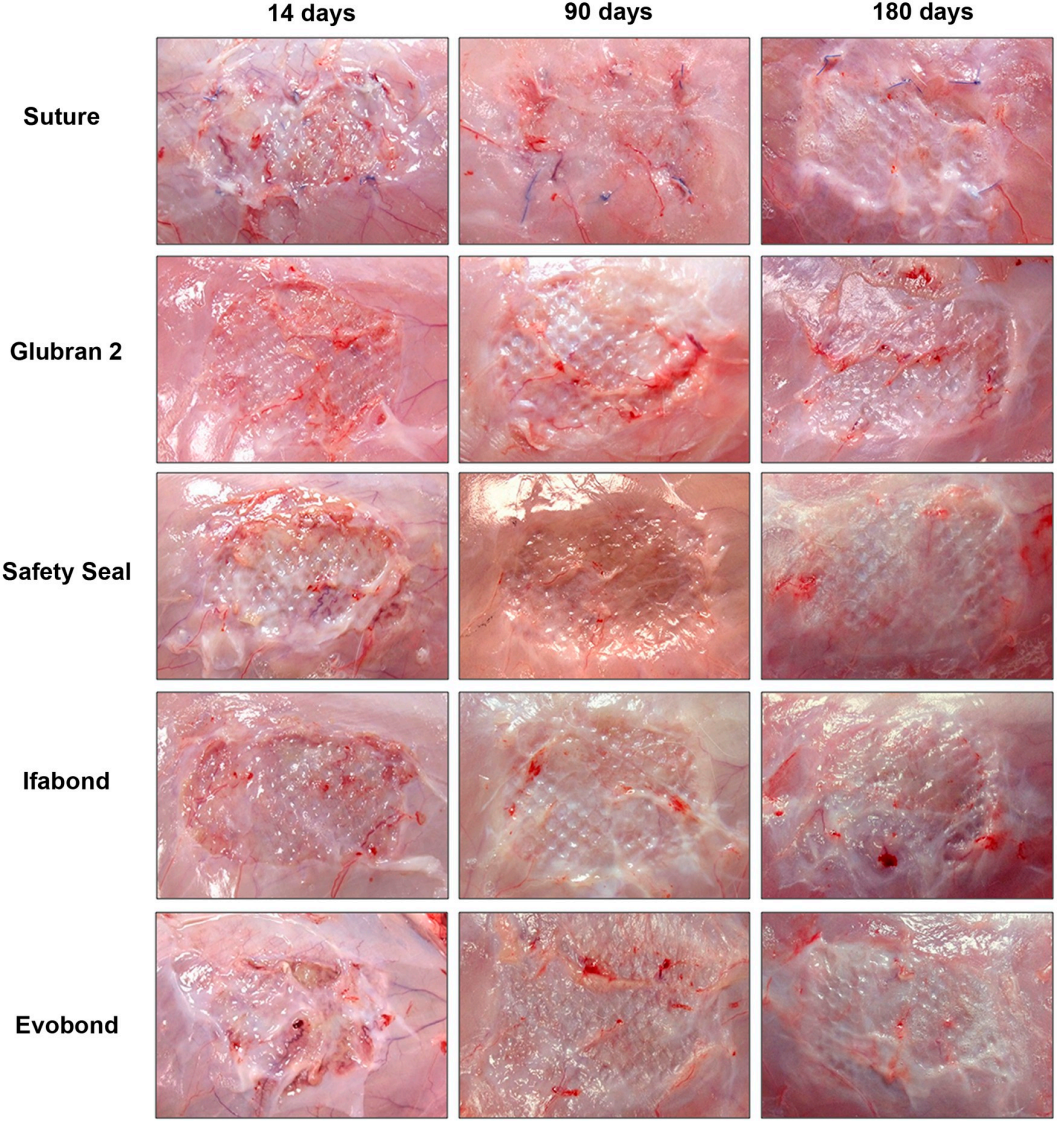
Macroscopic appearance of the neoformed tissue integrating the mesh. Images of the optimal integration of the Optilene Mesh Elastic polypropylene mesh in the different study groups. Aspect of the meshes fixed with sutures or with the different cyanoacrylate tissue adhesives (Glubran 2, SafetySeal, Ifabond and Evobond) 14, 90 and 180 days postimplantation. An animal with some encapsulation was shown in the image in the Evobond group at 14 days.

Tissue integration of the mesh with the neoformed tissue was only compromised in cases that showed any complications (Fig 2). The animals without complications showed good mesh integration, and all their prostheses were consolidated at 14 days post-implantation. This integration into the host tissue was consolidated over time, being complete at 90 days and 180 days (Fig 2).

### Morphological studies

**Fig 3:**
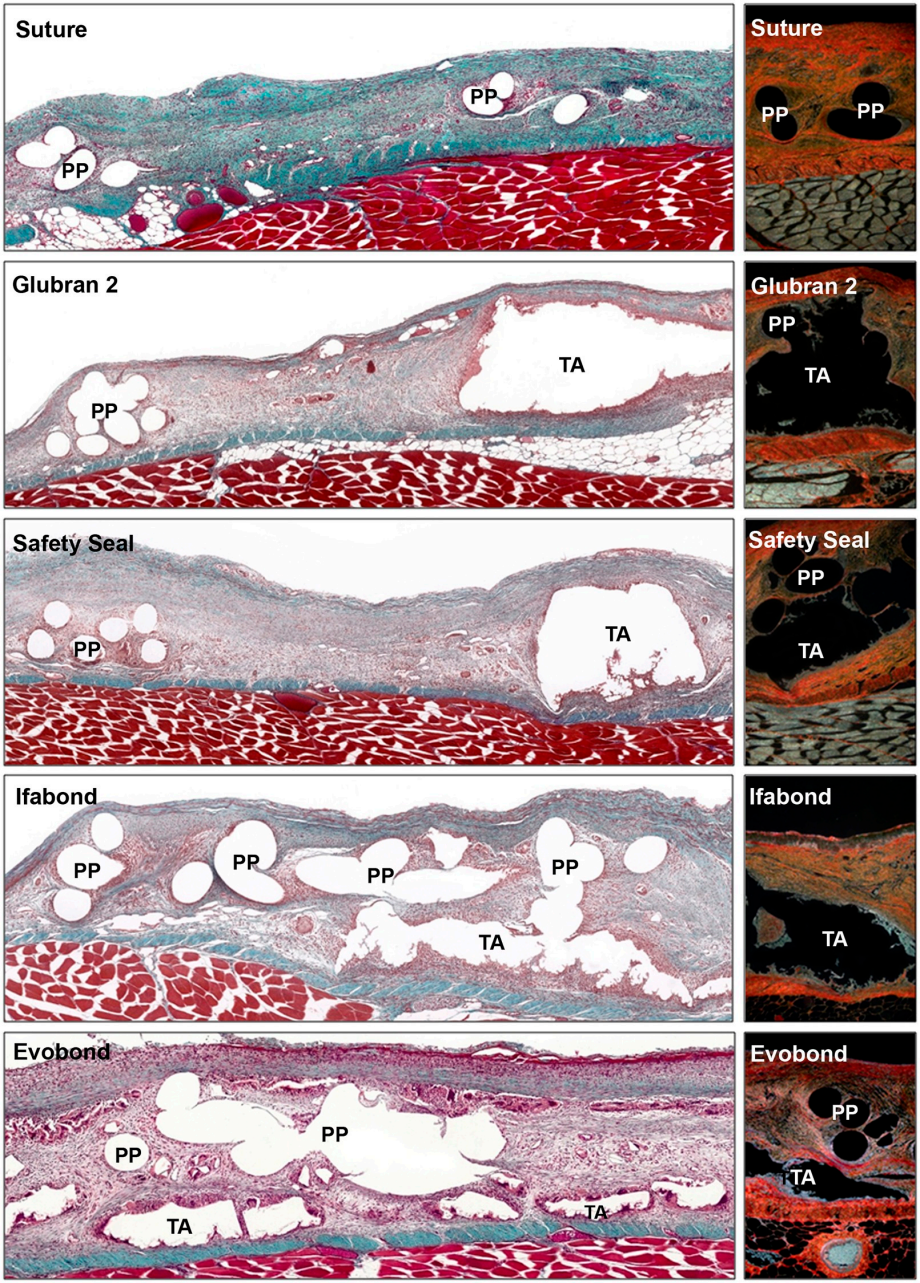
Morphological studies of the implanted meshes at 14 days. Light microscopy images of the large pore polypropylene mesh, fixed with stitches or with the different cyanoacrylates (Glubran 2, SafetySeal, Ifabond and Evobond), 14 days after implantation. *Left images*: Masson’s trichrome staining panoramic images of the implant meshes (100x magnification) show the space between the filaments and knots of the mesh in the control group), and in the adhesive groups, it is possible to see the area where the cyanoacrylate is fixing the mesh. *Right images*: sirius red staining images (100x magnification). Type I collagen is seen in red, and type III collagen is seen in yellow-green. *(PP*: *polypropylene mesh filament; TA*: *tissue adhesive)*.

Fourteen days after surgery (Fig 3), all prostheses fixed with stitches and with the different cyanoacrylates were already well integrated into the host tissue. The neoformed connective tissue observed in all the groups was infiltrated between the mesh polypropylene filaments, it filled all the spaces, and it surrounded the places where the cyanoacrylate was located. A more dense neoformed connective tissue, richer in collagen type I fibers, was observed in the control group with respect to the other groups, where a loose, newly formed tissue with predominance of type III collagen (immature) was developed. In the cyanoacrylate groups, adhesive infiltrates were observed between the mesh filaments. Inflammatory cells were found with light microscopy, especially in the Evobond cyanoacrylate group, during this study period. These inflammatory cells were in the areas near the mesh, surrounding the polypropylene filaments in the suture control group, whereas in the cyanoacrylate groups, these inflammatory cells appeared in a larger quantity and surrounded the place where the adhesive was located, isolating the cyanoacrylate from the neoformed connective tissue.

**Fig 4:**
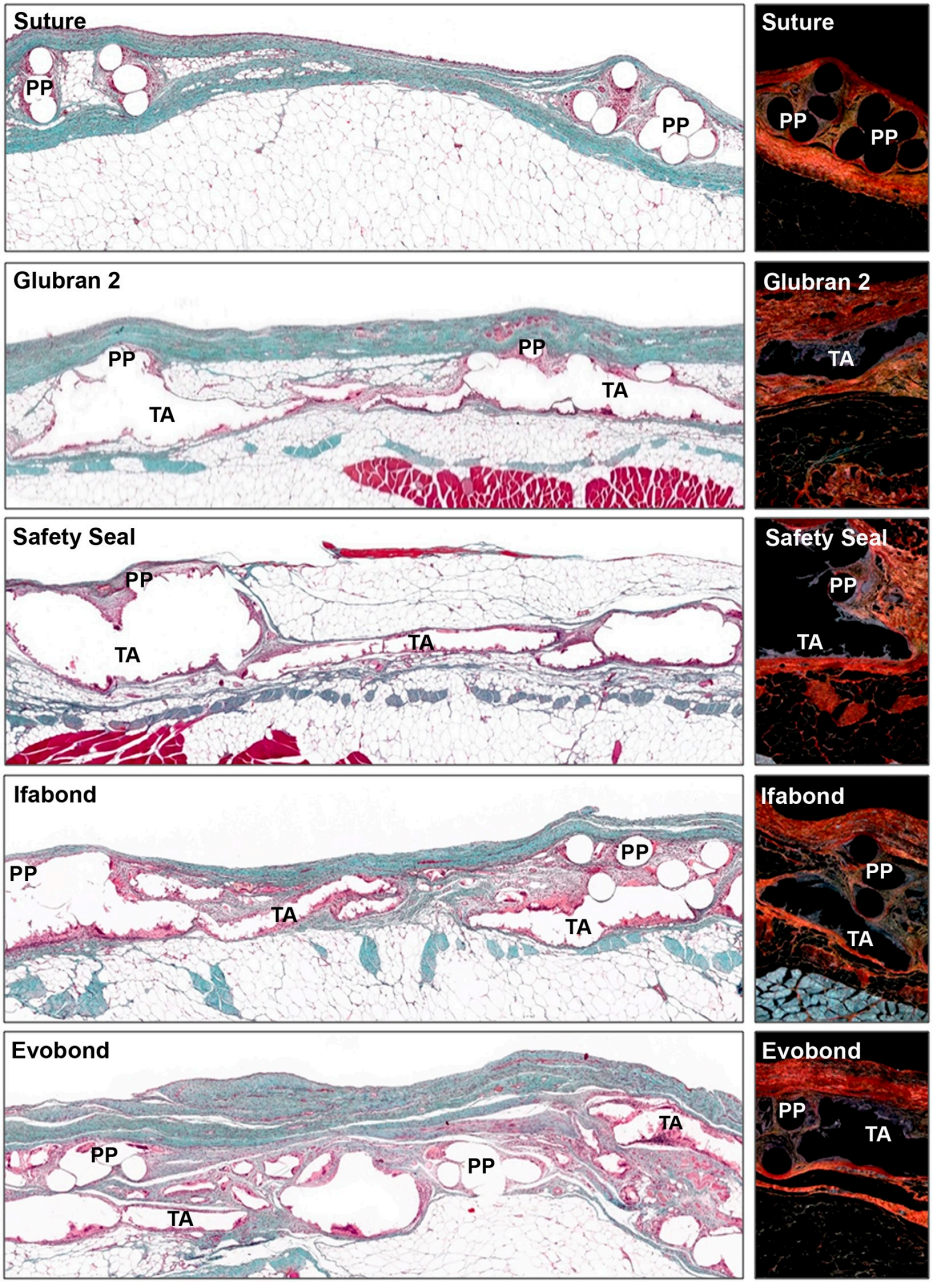
Morphological studies of the implanted meshes at 90 days. Light microscopy images of the large pore polypropylene mesh, fixed with stitches or with the different cyanoacrylates (Glubran 2, SafetySeal, Ifabond and Evobond), 90 days after the surgery. *Left images*: Masson’s trichrome staining panoramic images of the implant meshes (100x magnification). *Right images*: sirius red staining images (100x magnification). Notice the increase in type I collagen at this study time in relation to 14 days. Type I collagen is seen in red, and type III collagen is seen in yellow-green. *(PP*: *polypropylene mesh filament; TA*: *tissue adhesive)*.

Ninety days postimplantation (3 months), all the different cyanoacrylates continued without visible degradation (Fig 4). The newly formed connective tissue showed increased infiltration of adipose tissue, mainly in the area closest to the remaining transverse muscle, opposite the subcutaneous tissue. In general, the connective tissue was more dense and had a larger amount of type I collagen (mature) in the control and cyanoacrylate groups, although a large amount of type III collagen was also found. There was also a high quantity of inflammatory cells around the tissue adhesive borders, showing the presence of a large number of inflammatory cells and giant foreign body cells.

**Fig 5:**
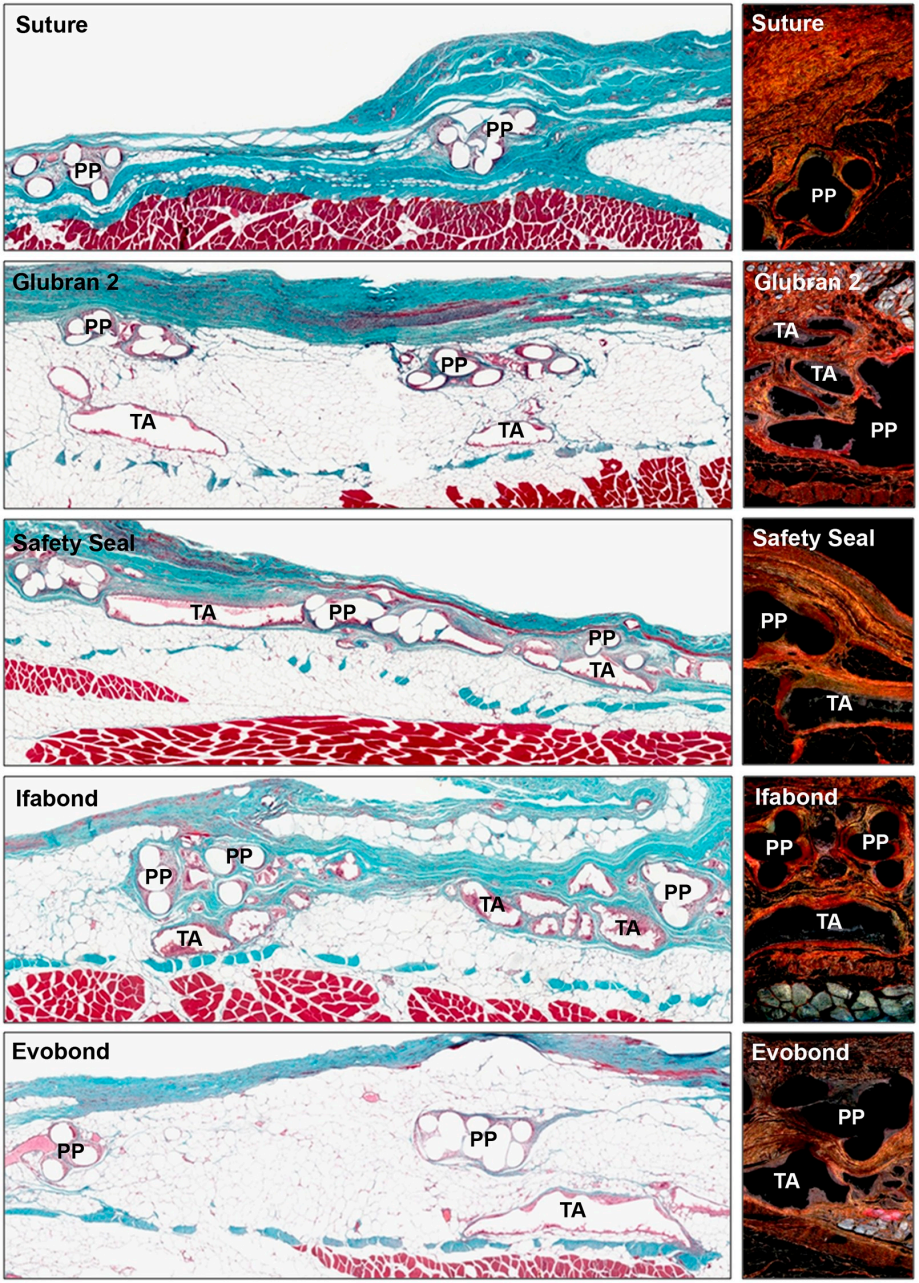
Morphological studies of the implanted meshes at 180 days. Light microscopy images of the large pore polypropylene mesh, fixed with stitches or with the different cyanoacrylates (Glubran 2, SafetySeal, Ifabond and Evobond), 180 days after implantation. *Left images*: Masson’s trichrome staining panoramic images of the implant meshes (100x magnification). *Right images*: sirius red staining images, where it is possible to appreciate collagen expression in the different implanted groups after 180 days. Type I collagen was the main collagen seen at this study time (100x magnification). Type I collagen is seen in red, and type III collagen is seen in yellow-green. *(PP*: *polypropylene mesh filament; TA*: *tissue adhesive)*.

Then, 180 days after surgery (Fig 5), the large amount of adipose tissue remained; in some groups, it even progressed towards areas of connective tissue near the prosthetic filaments and cyanoacrylate residues, relegating the connective tissue to areas close to the subcutaneous area. During this study period, the different cyanoacrylates showed no visible sign of degradation. The predominant collagen in the suture control group was type I collagen, which was very compacted around the polypropylene filaments. A large amount of type I collagen was also found in the different cyanoacrylate study groups, although a larger quantity of type III collagen was seen in the Evobond adhesive group. Inflammatory cells still surrounded the adhesives.

### Macrophage response

In general, during the different study periods, suture fixation (control group) showed macrophages located around the polypropylene filaments in a moderate way, while in the different cyanoacrylate study groups, macrophage cells were found around the adhesive in a more intensive way. The percentage of macrophage cells observed in the suture control group was significantly lower (all p<0.001) than that observed in any of the cyanoacrylate study groups, at any of the 3 different study times.

Fourteen days after prosthesis implantation, a significantly increased number of RAM11-positive cells were counted in the Glubran 2 group compared to the other three cyanoacrylates (p<0.01). In contrast, the groups of cyanoacrylates that showed the lowest values were SafetySeal and Evobond, which showed similar percentages to each other and were significantly decreased compared to the Ifabond adhesive (p<0.05).

After 90 days, values of labeled macrophages increased significantly in the Evobond tissue adhesive group compared to those after 14 days (p<0.05), while in the Glubran 2 and Ifabond cyanoacrylate groups, these levels significantly decreased (p<0.05), staying similar to the SafetySeal group. Only the Evobond group showed significantly increased percentages of macrophages compared to the other cyanoacrylates (p<0.001).

**Fig 6:**
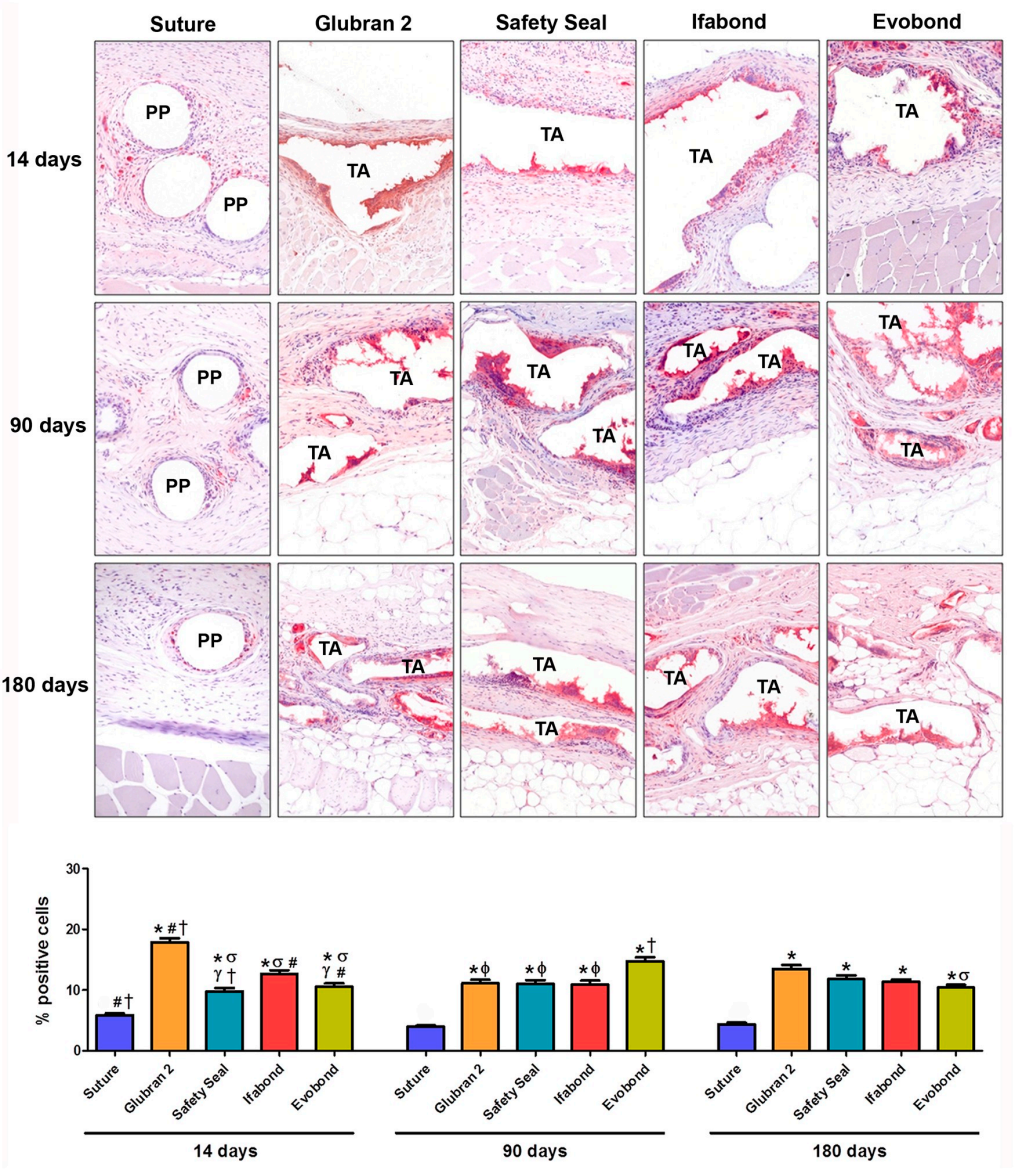
Foreign-body reaction analysis. Immunohistochemical labeling of rabbit macrophages (red) using the RAM11 monoclonal antibody in all the study groups (suture, Glubran 2, SafetySeal, Ifabond and Evobond) and during the 3 study periods (14, 90 and 180 days after the surgery). *Upper panel*: light microscopy micrographs of RAM11 labeling (200x magnification). *Bottom panel*: percentage of RAM11-positive cells recorded for each study group and study time. (* p<0.*001 vs*. *suture; σ p<0*.*01 vs*. *Glubran 2; γ p<0*.*05 vs*. *Ifabond; ϕ p<0*.*001 vs*. *Evobond; # p<0*.*05 vs*. *90 days; † p<0*.*05 vs*. *180 days)*. *(PP*: *polypropylene mesh filament; TA*: *tissue adhesive)*.

After 180 days, positively labeled macrophage values remained constant in the Glubran 2, SafetySeal and Ifabond groups against the values obtained at 90 days, whereas in the Evobond cyanoacrylate group, this percentage decreased significantly (p<0.05), becoming the lowest value of all cyanoacrylates at this time and showing a significantly lower value than the Glubran 2 cyanoacrylate (p<0.01). (Fig 6).

### Gene expression of collagens (qRT-PCR)

Overall, collagen 1 and collagen 3 mRNA expression levels were significantly increased in all the groups in the short term compared to the medium (p<0.001) and long term (p<0.001).

**Fig 7:**
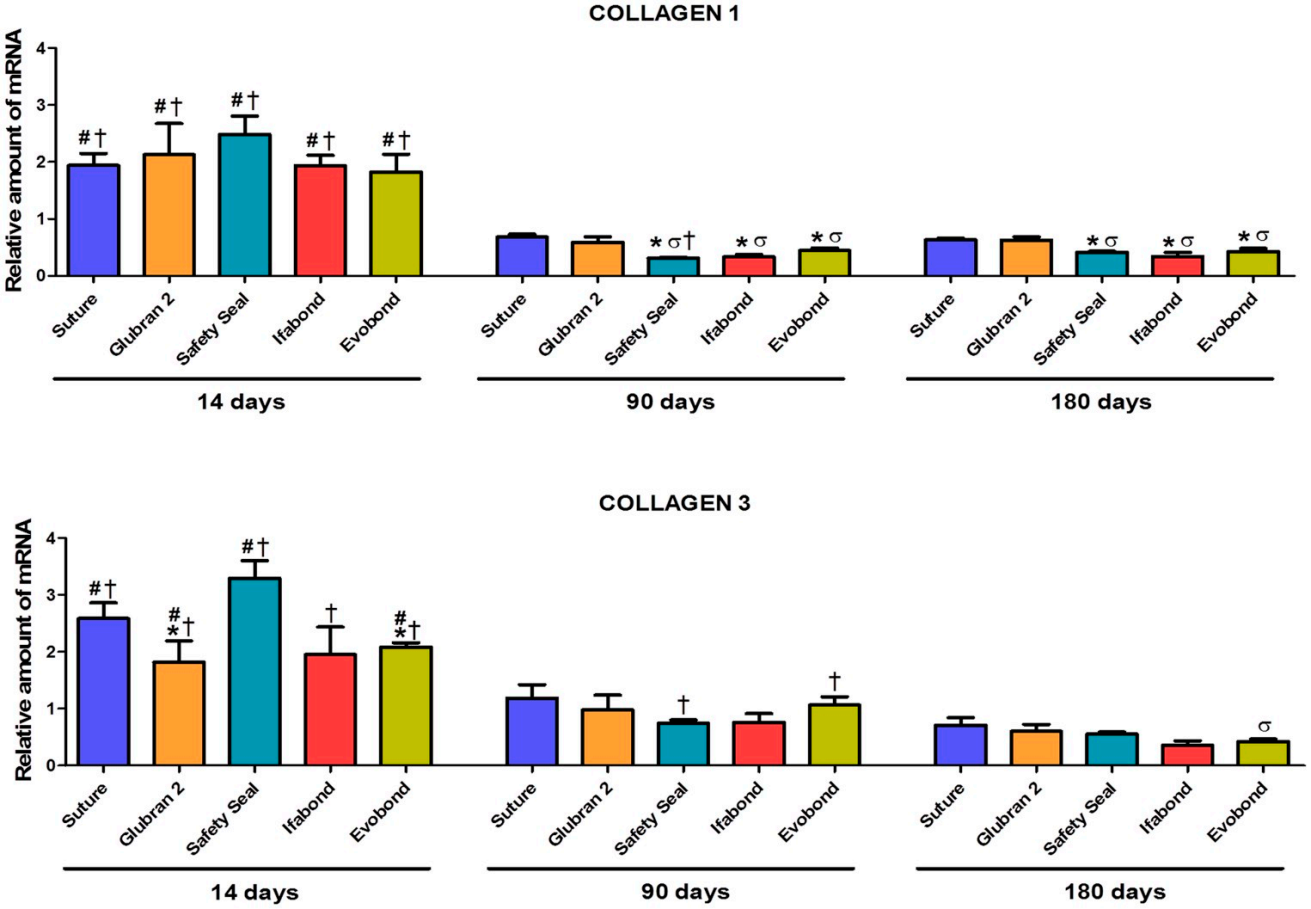
Relative collagen 1 and 3 mRNA expression levels determined by qRT-PCR in the implant areas. Relative collagen 1 mRNA expression (upper) and collagen 3 mRNA expression (lower) in the different study groups. Gene expression was normalized to the expression recorded for the reference gene GAPDH. *(Collagen 1*: * *p<0*.*01 vs*. *suture; σ p<0*.*05 vs*. *Glubran 2; # p<0*.*001 vs*. *90 days; † p<0*.*001 vs*. *180 days) (Collagen 3*: * *p<0*.*05 vs*. *Safety Seal 14 days; σ p<0*.*05 vs*. *Safety Seal 180d; # p<0*.*001 vs*. *90 days; † p<0*.*001 vs*. *180 days)*.

Collagen 1 mRNA expression levels 14 days after surgery were similar in all the study groups, without any significant differences among them. At 90 days postimplantation, collagen 1 mRNA significantly decreased compared to at 14 days, and this result was maintained at 180 days after surgery. However, at 90 and 180 days, significantly higher levels of collagen 1 gene expression were observed both in the suture group (p<0.01) and in the Glubran 2 (p<0.05) cyanoacrylate group versus the other cyanoacrylate tissue adhesives (SafetySeal, Ifabond and Evobond) (Fig 7).

Collagen 3 gene expression at 14 days postimplantation was increased in the SafetySeal cyanoacrylate group compared to the Glubran 2 and Evobond cyanoacrylate groups (p<0.05). In general, the expression of collagen 3 was increased at 14 days, significantly decreased at 90 days, and remained steady or slightly decreased at 180 days (Fig 7). At 180 days, significant differences were observed only between the SafetySeal and Evobond groups (p<0.05).

### Biomechanical analysis

**Fig 8:**
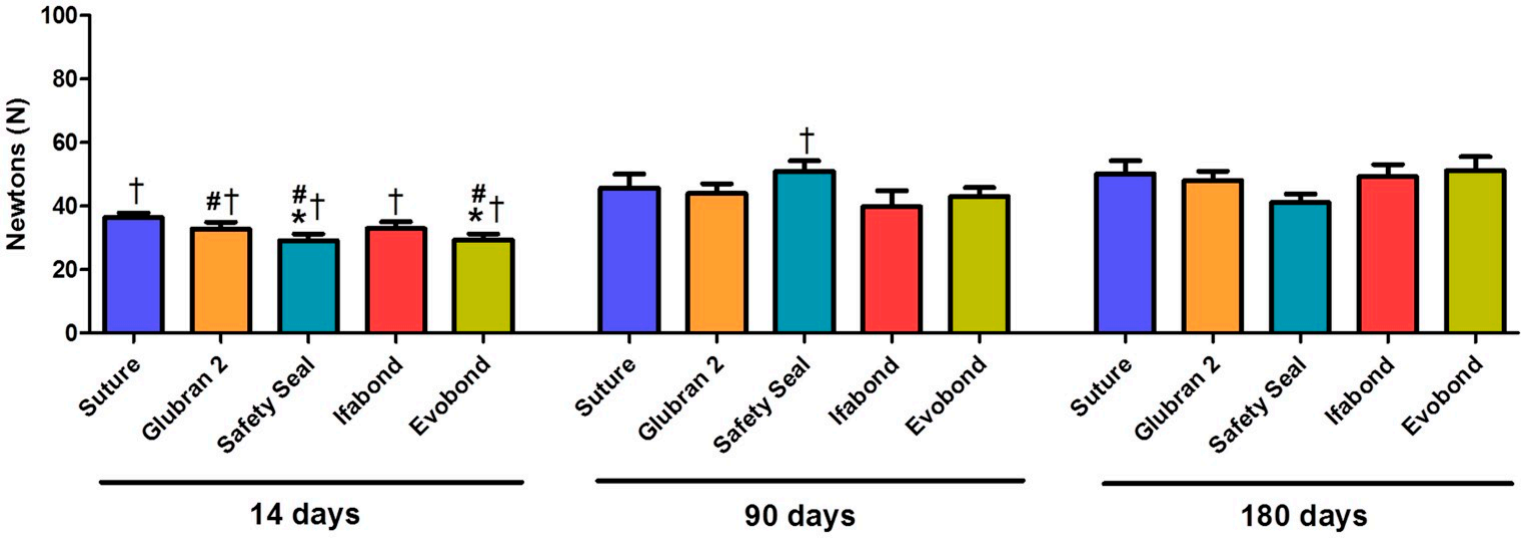
Biomechanical analysis. Maximum strength values for the different groups in newtons (N), recorded at the different study times (14, 90 and 180 days after the surgery). (* p<0.*05 vs*. *suture 14 days; # p<0*.*05 vs*. *90 days; † p<0*.*05 vs*. *180 days)*.

At 14 days, the Safety Seal and Evobond groups showed significantly decreased in the maximum biomechanical strength (p<0.05) with respect to the control group, but no significant differences were found for the other cyanoacrylate groups (Fig 8). These results are also reflected in the load-stretch curves for each animal and study group in the short term (S1 Fig).

In a general way at 90 days postimplant the maximum strength values increased in all study groups compared to the short term. This increase was statistically significant in the case of Glubrand, SafetySeal and Evobond (p<0.05). No significant differences were seen between the prostheses fixed with sutures and cyanoacrylates at this study time (Fig 8). The results of the load-stretch curves for each animal and study group in the medium term, were showed in S2 Fig.

Maximum strength values were increases over time in all the groups, in a non-statistically significant way, at long term (180 days) except Safety Seal that decreased slightly compared to 90 days (p<0.05). No significant differences were seen between the different groups 180 days after surgery (Fig 8). The results of the load-stretch curves for each animal and study group in the long term, were showed in S3 Fig.

## Discussion

In recent years, there has been a renewed interest in tissue adhesives, given the advantages of their application and the extension of their indications in the field of surgery [24]. One of the applications of TAs is in hernia surgery, where tissue adhesives are used to fix prosthetic materials. The use of sutures for the same purpose, both absorbable and nonabsorbable, has been subject to clinical reviews because some patients report postoperative pain, which has been attributed to nerve entrapment caused by the sutures themselves [8]. In clinical practice, some authors [13, 25, 26] have reported better comfort and less postoperative pain after hernioplasty using tissue adhesives to fix the mesh.

The continuous evolution of synthetic tissue adhesives requires the performance of preclinical studies that analyze aspects of the reparative process, foreign body reaction and tolerance of the adhesives. For this reason, we compared several cyanoacrylates, all of them long-chain, and therefore with a supposed low toxicity and good tolerance by the recipient tissue [27, 28]. The surgical model used was the creation of a partial tissue defect in the antero-ventral abdominal wall of the experimental animal, replacing the tissue with a LW prosthetic material and fixing it well with the tissue adhesive in the form of drops or with stitches (as a control) to achieve conventional prosthetic fixation.

The unconditional defense of LW prostheses [17, 29], which our group agrees with, is based on their ability to induce better adaptation to host tissue mimicking native tissue as described in detail previously [30], causing less fibrosis and inflammatory responses than high-density materials. Fixation using tissue adhesives, as a replacement for conventional sutures, may improve the process of hernia repair and the tissue trauma even more.

After the characterization of the abdominal muscle, as a prerequisite step to modeling hernia surgery [31, 32], the mechanical response of non-implanted surgical meshes with different pore sizes has been addressed [18]. In this respect, our group has made contributions in the field of biomechanics showing that PP-LW meshes such as Optilene [30] displayed a mechanical response similar to healthy muscle. Numerical simulations models of reconstructed herniated human abdominal wall based on abdomen geometry obtained by magnetic resonance imaging support this theory, as they have shown that these LW meshes showed the closest deformation to the natural distensibility of the abdomen [33]. Optimal clinical results in abdominal hernia repair strongly depend on a perfect match between the mechanical properties of the abdominal wall and those of the biomaterial used for repair in the long term [34].

The functional and morphological properties of HW versus LW PP meshes have also been addressed by several authors [35, 36], who concluded that a lower amount of foreign material implanted with an LW resulted in better abdominal wall compliance and that physiological abdominal wall compliance could be achieved in the long term after LW mesh implantation.

We have evaluated early host tissue incorporation of several PP LW meshes used to repair abdominal wall defects [21, 37]. We showed that large-pore meshes induced the genetic overexpression of collagen types 1 and 3 in the neoformed connective tissue, more collagen type III protein deposition and its faster replacement by collagen I, the main fibrillar extracellular matrix protein responsible for the mechanical support of the tissue. Indeed, it was the prosthesis chosen in the present study, Optilene, that showed, in the short term, the highest value of tissue resistance to traction, which would justify its support over small-pore prostheses and its choice for this study. In agreement with our observations, different authors [38, 39] also noted that LW prostheses showed a larger resistance at 30 days postimplantation than did HW meshes.

The clinical use of LW prostheses is also justified by different studies. Around eight hundred patients were analyzed in a multicenter randomized controlled trial comparing LW vs HW meshes in primary inguinal hernia repair. There was no difference in the complication rate or recurrence between LW and HW meshes, and LW significantly reduced the incidence of pain and favorably affected the perceived quality of life 6 months after implantation [40]. Other authors [41] showed similar results in a meta-analysis that pooled the effects of outcomes of 1002 patients enrolled into 5 comparative trials for hernia repair, highlighting that the use of an LW mesh seems to be associated with less chronic pain and no increase in recurrence and other postoperative complications.

Despite all their advantages, some authors affirm that the LW PP meshes with wide pores have sometimes caused problems in the clinic at the time of fixation due to sliding of the suture between the pore itself and the recipient tissue [42,43]. This issue can be a cause of disinsertion of the mesh and recurrence of the hernia process. This fact led us to investigate what would happen when, instead of sutures, a tissue adhesive was applied. To this end, we studied tissue integration and biomechanics at different study periods. We believe that very short study periods can give us information about the immediate behavior of the tissue adhesive, while the intermediate and long durations can provide information on the evolution of the foreign body reaction, bio-degradation of the tissue adhesive and its mechanical resistance.

Our macroscopic results showed no loss of mesh insertion or hernia recurrence in any of our groups, despite the use of large-pore meshes and cyanoacrylate fixation. Tissue integration of the mesh with the neoformed tissue was only compromised in cases showing complications, such as seroma or encapsulation, which were rare in our study. The rest of the animals showed an optimal host tissue integration at all study times and had good long-term mechanical behavior.

Maximum strength values were increases over time in all the groups, all groups showed a statistically significant increase between 14 and 180 days. This is a very important result of the biomechanical study since it confirms the prosthetic integration of the mesh by the host tissue and the effectiveness of the different CAs in the fixation. The high maximum strength value found in Safety Seal group at 90 days seems that the values of this group have deviated a bit, since at 180 days it can be seen how it decreases significantly. However, the most important fact from our point of view is that at both 90 and 180 days values are similar in the different groups, without showing significant differences among them nor with the conventional method of prosthetic fixation. Thus, these adhesives are a good and less traumatic option, even in the fixation of large-pore prostheses, which are susceptible to loss of insertion of the mesh. Thus, these adhesives are able to replace conventional suture techniques with reliability.

The suitability of these tissue adhesives in the fixation of high-density PP prostheses has already been tested by different authors and by our group in previous preclinical studies [22, 28, 44–46], which showed proper fixation and effective biocompatibility. The main problem of the CAs has turned out to be the general increase in the macrophagic response with respect to the sutures. We observed a maximal macrophage response in the short term, although the percentage of positively labeled cells decreased over time. Regarding the RAM11 expression, similar results at 14 days have been observed using HW meshes [28], in which Glubran and Ifabond showed greater values than octyl.

This larger number of inflammatory cells in the cyanoacrylate groups is not very important as to delay the maturation process of the neoformed tissue since, as our results show, this tissue is undergoing a progressive collagenization, increasing over time the collagen type I, the key extracellular matrix protein in the mechanical support of tissue. This fact is in complete agreement with the results obtained for biomechanical strength: a significant increase in all the groups in the long term. In addition, we must also take into account that the macrophage identification technique used in this study does not differentiate M1 macrophages, which promote inflammation and tissue injury, from M2 macrophages, which act to resolve inflammation and promote tissue reconstruction; thus, some of these identified cells may be of the reparative type.

Therefore, all the results obtained in our study indicate that all the chosen cyanoacrylates, despite inducing a significant increase in macrophage response versus sutures, showed optimal host tissue integration and long-term mechanical behavior; thus, they might be good choices in LW-PP mesh hernia repair.

As with any experimental study, our results need to be interpreted with caution, and our study is not without limitations. We are aware of the restrictions of our study, including the animal model and the insufficient study time required until the complete degradation of the cyanoacrylates. In our experience, although the rabbit model has provided optimal results in terms of tissue repair, immune responses and biomechanical behaviors, it is necessary to transfer the model to human clinical practice. To complement this study, the behavior of these adhesives in fixing LW meshes needs to be assessed in humans to confirm the behavior observed in the rabbit.

Despite these limitations, the consistency of our observations in this preclinical rabbit model demonstrates the advantageous characteristics of the low-density prosthesis together with the additional advantages offered by cyanoacrylate-based tissue adhesives in terms of ease and fast application in clinical practice. These traits minimize tissue damage and pain after hernioplasty, which would increase the quality of life of the patient.

## Supporting information

S1 Fig Load-stretch curves at 14 days. Load-stretch curves for each animal and study group in the short term, 14 days after implantation. Load is represented in newtons (N) and elongation in millimeters (mm).
(TIF)

S2 Fig Load-stretch curves at 90 days. Load-stretch curves for each animal and study group in the medium term, 90 days after implantation. Load is represented in newtons (N) and elongation in millimeters (mm).
(TIF)

S3 Fig Load-stretch curves at 180 days. Load-stretch curves for each animal and study group in the long term, 180 days after implantation. Load is represented in newtons (N) and elongation in millimeters (mm).
(TIF)
