# Supplementary figures and images for: Pre-clinical assay of the tissue integration and mechanical adhesion of several types of cyanoacrylate adhesives in the fixation of lightweight polypropylene meshes for abdominal hernia repair
Source: PLoS One. 2018 Nov 2;13(11):e0206515. doi: 10.1371/journal.pone.0206515 (PMC6214531; doi:10.1371/journal.pone.0206515)

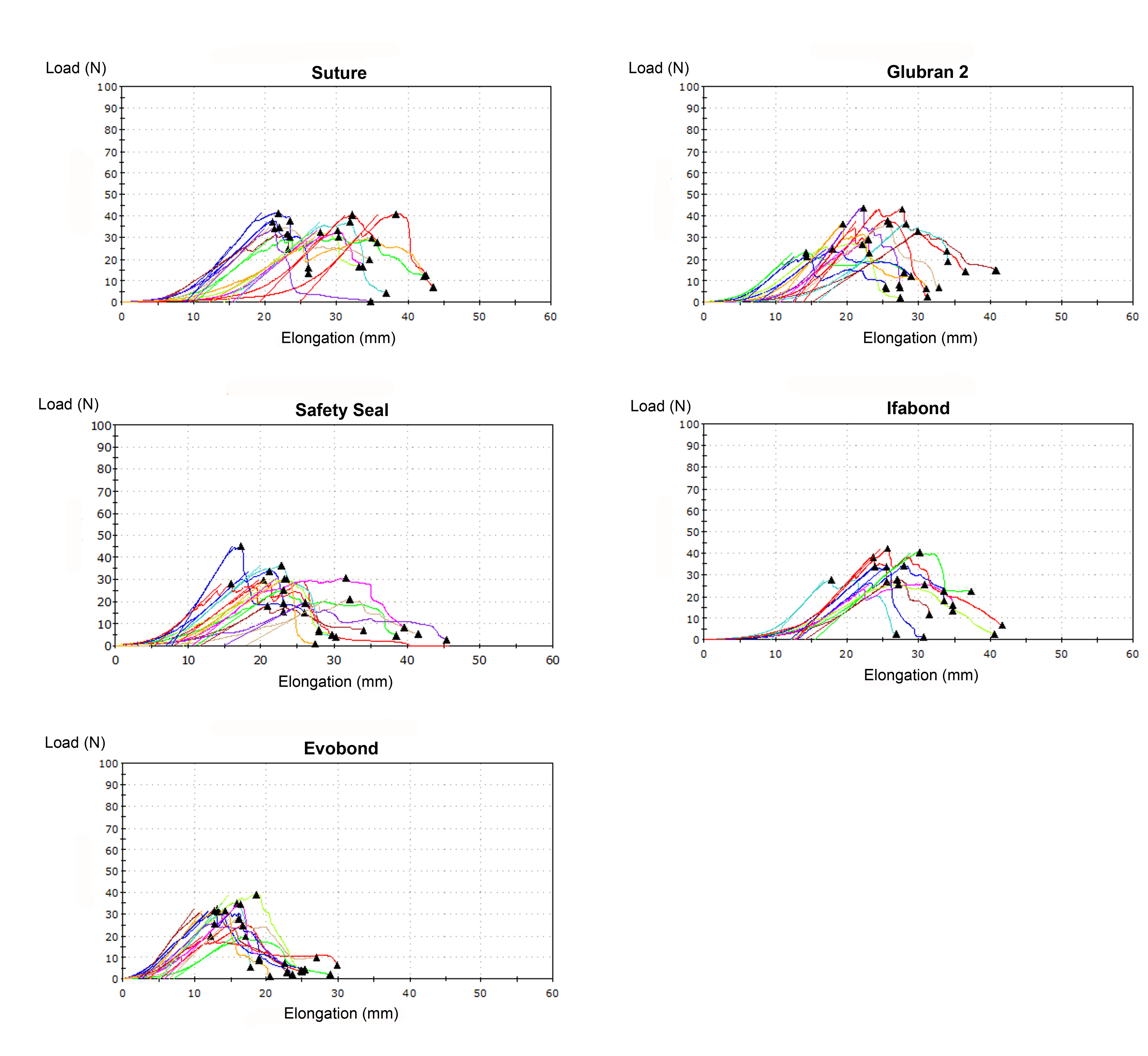

Supplement: S1 Fig — Load-stretch curves for each animal and study group in the short term, 14 days after implantation. Load is represented in newtons (N) and elongation in millimeters (mm). (TIF) [file pone.0206515.s001.tif]

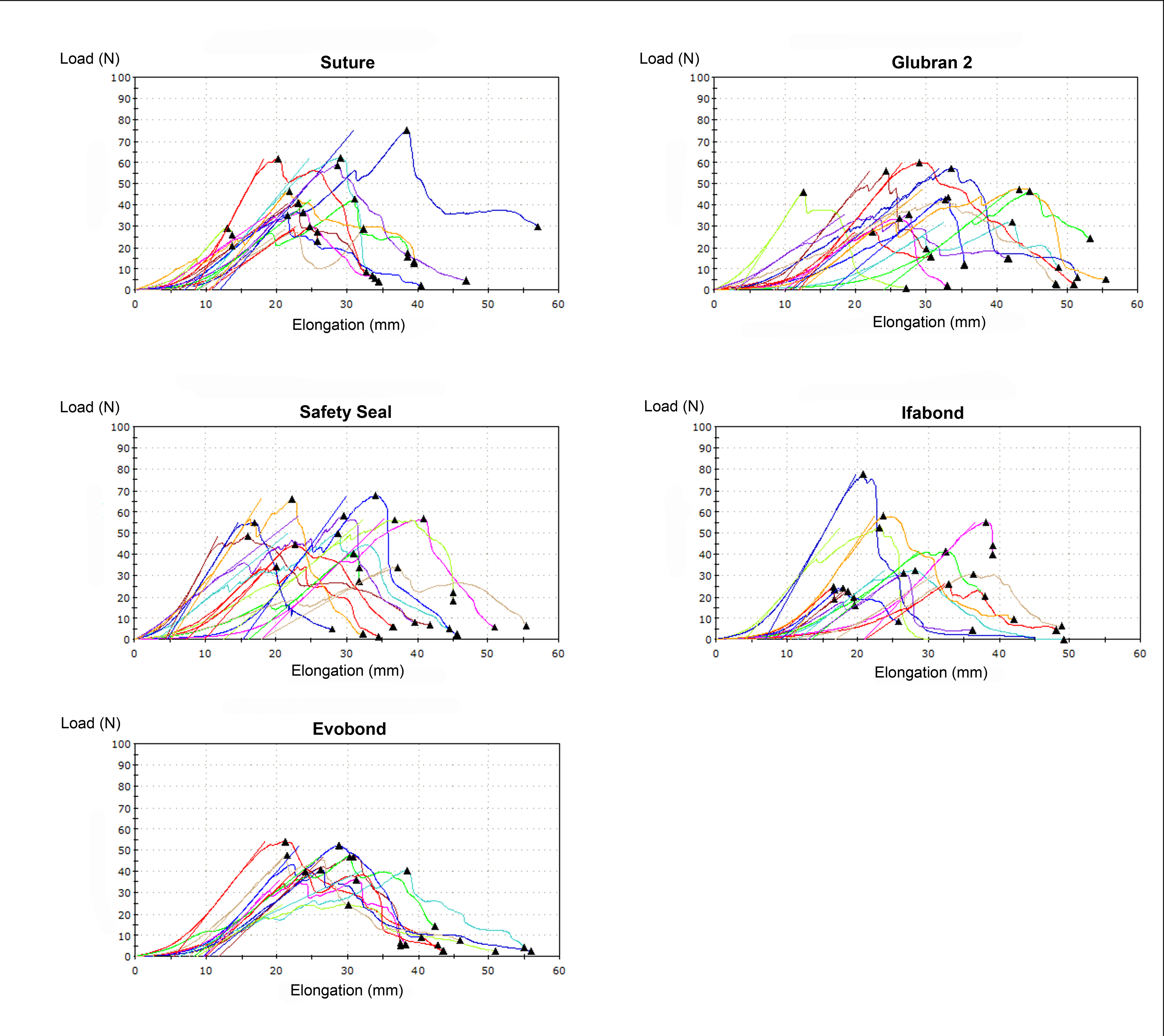

Supplement: S2 Fig — Load-stretch curves for each animal and study group in the medium term, 90 days after implantation. Load is represented in newtons (N) and elongation in millimeters (mm). (TIF) [file pone.0206515.s002.tif]

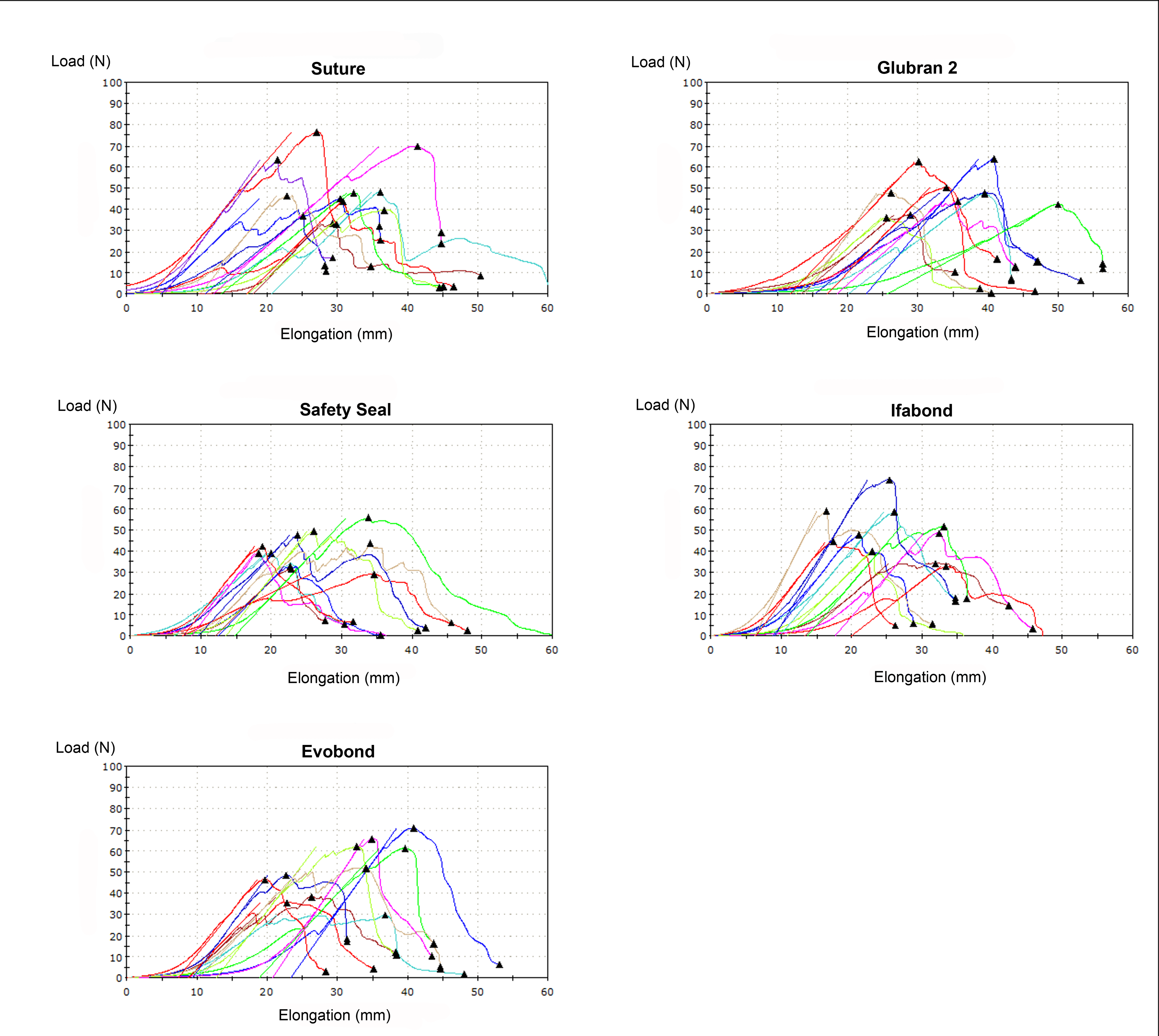

Supplement: S3 Fig — Load-stretch curves for each animal and study group in the long term, 180 days after implantation. Load is represented in newtons (N) and elongation in millimeters (mm). (TIF) [file pone.0206515.s003.tif]
